# A Model for Organizational Entrepreneurship with Organizational Culture Approach in Iran's Teaching Hospitals

**DOI:** 10.4314/ejhs.v31i2.25

**Published:** 2021-03

**Authors:** Fatemeh Rasooly Kalamaki, Ghahraman Mahmoudi, Jamshid Yazdani Charati

**Affiliations:** 1 Department of Health Services Management, Sari Branch, Islamic Azad University, Sari, Iran; 2 Hospital Administration Research Center, Sari Branch, Islamic Azad University, Sari, Iran; 3 Biostatistics department, health science research center, addiction institute, Mazandaran university of medical sciences, sari, Iran

**Keywords:** Entrepreneurship, Organizational Entrepreneurship, Organizational Culture, Hospital

## Abstract

**Background:**

One of the most basic tools and strategies for developing new ideas and entrepreneurship is establishing the entrepreneurial culture in the organization. Thus, the purpose of this study was to examine the effect of organizational culture on entrepreneurship of district 1 teaching hospitals in Iran.

**Method:**

The study was applied in terms of purpose and descriptive-analytical of survey type in terms of nature that was conducted in 2019 on 946 staff members of district 1 teaching hospitals in Iran using census sampling method. The measurement tools used were standard questionnaires of Margaret Hill Entrepreneurship and Edgar Schein Organizational Culture. Pearson correlation coefficient was used to determine the relationship between organizational culture and organizational entrepreneurship. Partial least squares (PLS) was used for structural equation modeling (SEM) and analyzed in Smart pls2 software.

**Results:**

The results showed that there is a significant direct relationship between the organizational culture and organizational entrepreneurship (r=0.94). Also, there was a relationship between the internal consistency component with organizational entrepreneurship (r=0.93), between the external compatibility component with organizational entrepreneurship (r=0.90). (p≤0.05)

**Conclusion:**

The results indicated a positive and significant relationship between organizational culture and its dimensions (internal consistency and external compatibility) with organizational entrepreneurship. Thus, making the decisions that concentrate on the organizational culture of the hospitals and taking steps to coordinate people's values and norms that promote organizational culture and enhance organizational entrepreneurship are recommended.

## Introduction

The move towards entrepreneurship is so important for success in today's changing world. Nowadays, entrepreneurship is considered as one of the tools of development since entrepreneurial people provide the context for success. Moreover, considering the rapid growth of new competitors and the emerging distrust in traditional methods, the need is felt for entrepreneurship in the organization. Regarding this, the dynamic organizations' duty is to discover and nurture creative and entrepreneurial individuals. Each organization requires the right structure and entrepreneurial people to nurture creative and innovative people (1). Organizational entrepreneurship happens in the organization and is a revolution in it trying to change and modernize the system within inside (2). Entrepreneurship is an opportunity-based way of thinking and acting (3).

Policymakers in Europe and America argue that more entrepreneurship is needed to reach higher levels of economic growth. Moreover, they believe that higher levels of entrepreneurship can be achieved through education, especially entrepreneurship education (4). An entrepreneur is someone who commits to run, manage and undertake the risk of economic activity (5). Organizational entrepreneurship promotes entrepreneurial behavior in an organization and involves the tendencies and activities that better the organization ability in face of risks and facilitates the hunt for opportunities and innovation (6). Entrepreneurship is an important factor for the success of an organization, especially in the world today. Previous studies have proposed entrepreneurial orientation as a significant factor for organizational success and profitability (7,8). Moreover, scholars state that to enter the market and enhance performance, the companies must have a strong entrepreneurial orientation so that the organizations with high levels of entrepreneurial orientation perform better than other organizations (9). Felgivora and Rodriguez state that the higher the level of entrepreneurial orientation in an organization, the greater the organization's attention to the market and strategic planning according to market demands and competitor movements (10).

Many experts have stated that common values derived from organizational culture have proven beneficial to the effort of more staff. Therefore, organizational culture has a positive effect on organizational efficiency regardless of its working context (11).

The necessity of attention to organizational culture is such that scholars believe that if an effective and sustainable change is to happen in an organization, the culture of that organization must be changed. In other words, the success and failure of the organizations should be sought in their culture (12). In a study Kiyd and Roselli concluded that organizational culture had a direct and positive effect on organizational entrepreneurship indices that include risk, innovation and pioneering (13).

In the organizations where entrepreneurial culture dominates, one can observe that the employees are the key element to success. The idea that entrepreneurs are the key to a country's economic growth and prosperity has been emphasized by leading experts like Schumpeter, Stevenson, and Jarillo (14). Organizational culture and management style are of the significant elements that affect the development of innovative and entrepreneurial behavior in organizations. According to Morrison, culture is very important in entrepreneurship as this concept determines people's attitudes towards establishing new entrepreneurial activity. The emergence of entrepreneurial culture has formed the formation of new organizations and small and independent companies with the hope of economic growth (15).

Monica (2006) conducted a study entitled “Organizational entrepreneurship as a way to develop the work and the quality of organizations' performance,” with the results showing that managers should shape organizational entrepreneurship culture as a central part encompassing all employees. Any kind of creativity should be made public fast, and creative people in the organization should be encouraged; the organization should devise ways to developing new services and products, which is very hard as it is all the responsibility of the manager. In a study entitled “Entrepreneurship process in organizations”, Zamptax et al. found that the employees receiving a high level of organizational support - when their organization values their being competent - trust more in their organization and their performance becomes better (16).

Felgivora and Rodriguez believe that the higher the level of entrepreneurial orientation in an organization, the greater the focus of the organization on the market and strategic planning (10).

Many scholars, such as Pinchat, Kuratko, et al., Lampkin, and Des have tried to consider the significance of organizational entrepreneurship as a growth strategy and as an effective tool to reach a competitive advantage (17–19). Moreover, efforts have been made to identify and specify empirical elements of corporate entrepreneurial activities through empirical studies (20). The results of such studies show that intra-organizational factors are closely related to the creation of the organizational entrepreneurial atmosphere, and one of the key components of the organizational entrepreneurship model has to do with the creation of an organizational culture that incorporates the core values of entrepreneurial culture like the motivating factors in the organization environment (21,22).

While everyone acknowledges the role of entrepreneurship, especially the importance of organizational culture in organizational entrepreneurship development, surveys show that entrepreneurship in the health sector of Iran has been less addressed so far. Therefore, despite the potential for entrepreneurship in this sector and the many intra-organizational and extracurricular opportunities that exist for the survival and promotion of healthcare organizations to cope with the overwhelming changes and fluctuations in the industry, no significant action has been taken in this field. In this regard, due to the diversity and multiplicity of service sector activities, especially healthcare services, it is possible to benefit from individual, group and organizational entrepreneurship to increase resource efficiency and effectiveness of the activity, and ultimately to improve quality and improve productivity. However, the main problem in this regard is the lack of common literature among managers and practitioners and the lack of deep knowledge of executives and planners on the concepts, themes, dimensions, and barriers of entrepreneurship. For this reason, entrepreneurship has not been exploited in its proper sense, and it has not been properly utilized in the health sector, which has the characteristics necessary for entrepreneurship.

## Method

This was a descriptive-analytical study applied in terms of purpose and cross-sectional regarding time, which was conducted in 2019 on the employees of Group 1 Planning Hospitals including public hospitals of universities (Mazandaran, Babol, Semnan, Golestan, Shahroud and Gilan). The hospitals were selected using clustered and randomized sampling methods, where overall 17 hospitals were selected. The population of the study was all managers (hospital managers and heads, supervisors, metron, head of nursing services and nursing experts and managers of health services) from clinical and paraclinical units, who were 946 people. This study was based on a census sampling method of all the staff of the public hospitals. Medical ethics code and study introduction letter were obtained from the relevant university for all the hospitals under study. In addition, the subject of the questionnaire was explained to those participating in the study with at least a bachelor's degree. Furthermore, informed consent was obtained, and the study subjects were assured of the confidentiality of their information. The individuals who were fully conscious and willing to participate were entered into the study. Moreover, the exclusion criteria were also the individuals' reluctance to continue the task and incomplete questionnaires. People who were fully conscious and willing to participate in the study entered the study and the exclusion criteria were unwillingness to continue working and deleting questionnaires without correct answers.

Conducted ethical considerations include: obtaining medical ethics code and a research introduction letter from the relevant university for all hospitals under study, explaining the subject of the questionnaire to those who participated in the study who had at least bachelor degree, and obtaining informed consent and the confidentiality of the questionnaires. Individuals who were fully conscious and had willingness to participate in the study were entered into the study and the exit criterion also included the reluctance of individuals to continue the task, and eliminating questionnaires that did not have the correct answer.

Data collection tools were standard questionnaires of organizational culture with 12 questions, in two dimensions of internal consistency and external compatibility (23) and organizational entrepreneurship of Margaret Hill with 32 questions, including organizational actions, individual attitude and entrepreneurial culture, each with 6 items, reward and flexibility status each with 5 items, and entrepreneurial leadership with 4 items (24). Validity and reliability of both questionnaires were verified by experts, and Cronbach's alpha coefficients of organizational culture questionnaire were 0.93 and entrepreneurship questionnaire 0.86. Both questionnaires were scored by 5-point Likert scores (very low = 1, low = 2, medium = 3, high = 4, very high = 5), and distributed in person among the population.

## Results

The results showed that 69% of the subjects were females while 31% were males. The distribution of respondents by education was 0.4% associate degree, 61.7% BS, 25.8% MS, 5.8% Ph.D., and 6.3% professional doctorate.

After extracting scores from organizational entrepreneurship questionnaires and organizational culture questionnaires in total 946 people, the relationship between the two components of organizational culture (i.e. internal consistency, and external compatibility) as well as the organizational culture as independent variable and organizational entrepreneurship as dependent variable were analyzed by Pearson correlation test.

Based on the results, it can be said that based on the results of Pearson correlation coefficient (with a significant level of Sig. ≤0.05), there is a significant direct relationship between the internal consistency and organizational entrepreneurship (r=0.93). Also, there was a relationship between the external compatibility with organizational entrepreneurship (r=0.90), and between organizational culture with organizational entrepreneurship (r=0.94).

To test the conceptual model of the research, the model analysis algorithm in the structural equation method of Smart PLS was used as follows, and the necessary analyses were performed to fit the measurement models and to fit the structural model. Cronbach's alpha is the classic measure of reliability and the index of internal sustainability assessment. Internal stability indicates the degree of correlation of a structure and its related indices, with a criterion higher than 0.6 indicating acceptable reliability.

To determine the reliability of each construct, in addition to the traditional Cronbach's alpha criterion, they use the more modern composite criterion (CR). The superiority of this criterion over the Cronbach's alpha coefficient is that the reliability of the structures is calculated not in absolute terms but in relation to the correlation of their structures with each other. Both criteria are used to better assess the reliability of both measures. Factor loadings were higher than 0.7 to confirm convergent validity.

The mean extracted variance (AVE), which is one of the main indices of convergence of the questionnaires for each variable was above 0.5 and finally, the third convergent validity condition (CR> AVE) was verified by the researcher.

Divergent validity tests in this study included the transverse load test and Fornell-Larcker test (25), which were performed and confirmed before implementing the structural model (inter-model). Therefore, the researcher was allowed to present the structural model with PLS. The modified structural model of the research is shown in Table 2. The structural model of the research was also significantly reviewed and confirmed by the researcher. The communality index was used to evaluate the model quality of each latent variable. The positive values of this index indicate the quality of the latent variables measurement model.

After fitting the measurement models, the PLS fits according to the data analysis algorithm in the Structural Model Research Method. In contrast to the measurement models in which the relationships between latent variables with explicit variables are considered, in analyzing the structural model of inter-relationships, the t-values of the present variables were analyzed together and the criteria of R^2^ significance coefficients for fitting the structural model were investigated.

**Figure 2:**
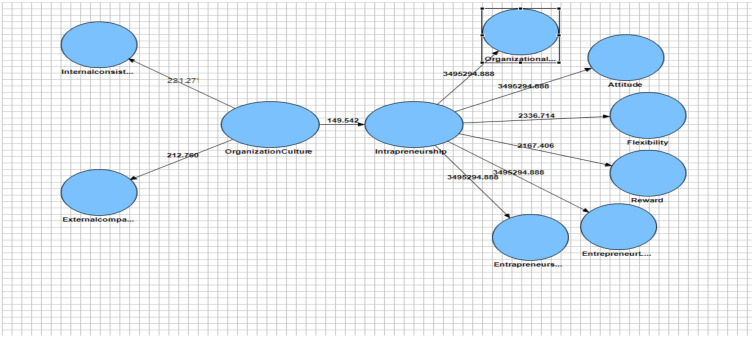
T-values for the structural part of the research model

Several criteria were used to evaluate the suitability of the structural model of the research, the first and most basic of which are the coefficients of Z, or t-values, and are represented by the bootstrapping command on the lanes. If the t-values are greater than 1.96, it indicates the accuracy of the relationship between the constructs and thus confirms the research hypotheses at a 95% confidence level. Figure 2 shows the t-values for the structural model evaluation. Given that all the numbers on the paths are above 1.96, this indicates that the paths are meaningful, that the structural model is appropriate and that all the research hypotheses are confirmed.

**Figure 1:**
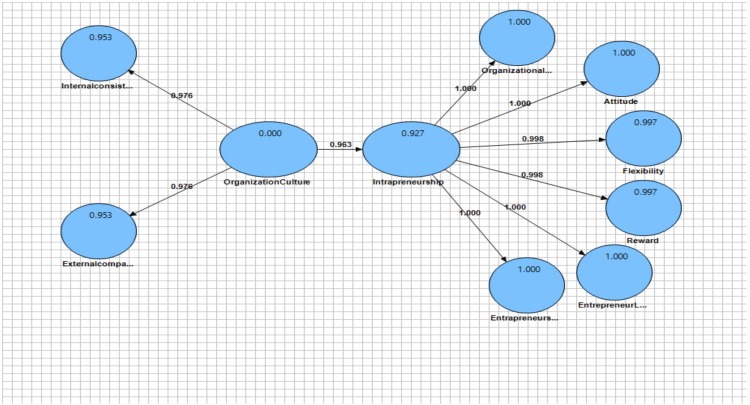
Path coefficient, R^2^ factor load values

The second criterion necessary to check the fit of the structural model is to examine the coefficients of determination of R^2^ for the present endogenous (dependent) variables of the model. This criterion was used to connect the measurement and structural components of structural equation modeling and to illustrate the effect of an exogenous variable on an endogenous one. Based on the conceptual model tested in Figure 1, and the numbers on the lines, it shows the path coefficient and the relationship between the present variables. It should be noted that the values of R^2^ are shown within the model circles and are calculated only for endogenous (dependent) of model structures and for exogenous structures, the value of this criterion is zero. Three values of 0.19, 0.33 and 0.67 have been introduced as criteria for model weak, medium and high (26). Three values of 0.19, 0.33 and 0.67 were presented as weak, medium and high for model fit (26). The values of the coefficient of determination can be seen in Table 1 and Figure 1. The R^2^ value for the organizational entrepreneurship variable was 0.57, for the entrepreneurial personality traits variable was 0.97, and for internal locus of control and achievement dimension was 0.99. Given these values, the criterion of the suitability of the structural model is confirmed.

**Table 1:** Mean extraction variance and composite reliability for the research variables

	AVE	Composite Reliability	R Square	Cronbachs Alpha	Communality	Redundancy
**Attitude**	0.92	0.99	1.00	0.98	0.92	0.92
**Entrepreneurship**	0.92	0.99	1.00	0.98	0.92	0.92
**Entrepreneur**	0.92	0.98	1.00	0.97	0.92	0.92
**Leadership**						
**External compatibility**	0.57	0.88	0.95	0.83	0.57	0.54
**Flexibility**	0.93	0.98	1.00	0.98	0.93	0.92
**Internal consistency**	0.57	0.88	0.95	0.84	0.57	0.54
**Intrapreneurship**	0.92	1.00	0.93	1.00	0.92	0.86
**Organization Culture**	0.54	0.93	0.00	0.92	0.54	0.00
**Organizational Verbs**	0.92	0.99	1.00	0.98	0.92	0.92
**Reward**	0.93	0.98	1.00	0.98	0.93	0.92

In accordance with the data analysis algorithm in the PLS method, after examining the fit of the measurement and structural models, the research hypotheses were tested by examining the Z-coefficients of the paths (t-values) and standardized factor loadings of the paths (Figure 2).

If the significance coefficient of each of the paths is more than 1.96, the corresponding paths at 95% confidence level and its related hypothesis are confirmed.

To check the significance of the path coefficient, the t-coefficients of each path are considered. Since the required t-value of each route is higher than 1.96, predicted routes are significant at a 95% confidence level. Therefore, the relevance of the present study is confirmed.

## Discussion

With a new and innovative view of entrepreneurship and its significance in hospitals as an organization providing health and treatment services, the study tried to examine and identify the relationships between organizational culture and its dimensions with organizational entrepreneurship and was designed and organized to find significant relationships and their values.

According to the results of Pearson correlation test, there is a significant and positive relationship between organizational culture in internal consistency with a focus on common language, the boundary between working groups in the organization, reward and punishment system, position and power relations within the system with organizational entrepreneurship in the studied hospitals (r = 0.93). This result is in line with those of Bojika et al, Yesil and Vakaya, Salimath and Cologne, and Paunovi and Dima (27–30).

The results showed a positive and significant relationship between organizational culture in terms of external adaptation, whose key components were mission and strategy, organizational goals, control tools and system with organizational entrepreneurship in the studied hospitals (r = 0.90). Concerning external adaptation of the organization, relying on external focus has a high degree of flexibility that provides the base for the growth of creativity and entrepreneurship, which is in line with the results of Yesil and Kaya and Naranjou et al. (28,31).

The results showed that focusing on these two dimensions of organizational culture (external compatibility and internal consistency) by considering the above-mentioned factors and components could illuminate the path towards entrepreneurship and betterment in organizations like the hospitals studied. In other words, cohesion of the intra-organizational behaviors with changes in the environment and external adaptation is necessary to enhance organizational entrepreneurship. Thus, one can understand that organizational entrepreneurship is in line with many organizational variables like organizational culture. According to the results of Pearson correlation test, there is a significant and positive relationship between organizational culture with organizational entrepreneurship in teaching hospitals (r = 0.94). It seems that entrepreneurship will be improved and vice versa if supported by entrepreneurial organizational culture.

This finding is supported by many researchers, such as Yildizet al. (32), Zhenget al.(33), Bojika et al (27), Hayton, Zahra, Bing, Triandis, Lounsbury, Altinay, Arz, Alpkan and Puhakka (34–42). For the hospital to tend towards entrepreneurship focus, there is a need for focus on their organizational culture and steps to be taken in line with the values and norms of individuals and employees with their norms and values.

Considering the direct relationship between organizational entrepreneurship and organizational culture, it seems that entrepreneurship is affected by organizational culture. By enhancing organizational culture, the weaknesses and the barriers that organizations face will be eliminated and organizational entrepreneurship will be enhanced. In other words, the results indicated that the more the organizational culture moves towards strengthening entrepreneurship and using tools for this purpose, the better the organization's entrepreneurial situation will become.

According to the present study, it is suggested that hospitals pay more attention to appointing managers who value entrepreneurship. They should identify and develop elements that can enhance organizational and personal entrepreneurial factors and improve the entrepreneurial spirit. Entrepreneurial education classes and workshops among hospital staff can also be more effective. Managers need to devote more resources to boost hospital staff entrepreneurship and creativity to keep up with the changing environment. In this way, making decisions that affect each of these two variables will improve and lead to an entrepreneurial organization. With proper planning in this regard, the factors affecting these two variables need to be identified and investigated in order to provide suitable solutions for a healthy and entrepreneurial organization.
